# Medical Students’ Attitudes to Mental Health and Psychiatry

**DOI:** 10.1192/bjo.2026.11311

**Published:** 2026-06-30

**Authors:** Ben Johnston

**Affiliations:** SE Trust, Downpatrick, United Kingdom

## Abstract

**Aims:**

To assess medical students’ attitudes towards mental health and psychiatry across three domains:

Psychiatry as a medical specialty.

Mental health patients.

Stigma in mental health.

**Methods:**

Medical students on placement in the Northern Trust and in the South-Eastern Trust (NI) were sent a survey to complete. The survey contained demographic data as well as 15 statements attached to a 5-point Likert scale, for students to select *strongly agree*, *agree*, *neither agree nor disagree*, *disagree*, or *strongly disagree*. Completed surveys were collected via a third party and forwarded to me for review, ensuring anonymity.

**Results:**

17 students completed the survey in full and their responses were analysed. Some statements had very consistent responses which produced a strong theme, as follows:

• 100% agree/strongly agree that ‘all medical students should complete a placement in psychiatry’.

• 100% disagree/strongly disagree that ‘psychiatrists are not real doctors’.

• 100% disagree/strongly disagree that ‘psychiatrists are less credible than doctors in other specialties’.

• 88% agree that ‘mental health conditions can be effectively treated’ – with 12% neither agreeing nor disagreeing. No students strongly agreed.

By contrast, a number of statements yielded a wide variety of responses with no clear theme, as follows:

• All five responses were selected (in various numbers) for the statement, ‘medical professionals treat patients with mental illness differently to those with physical illness’.

• All five responses were also selected for ‘patients with mental illness receive poorer care than those with physical illness’.

• All five responses were selected for “I worry that seeking help for my own mental health might negatively impact on my future career’.

• For the statement, ‘I would feel uncomfortable being alone with a patient who has a serious mental health issue’, 41% disagreed while 47% agreed/strongly agreed.

**Conclusion:**

Some clear, consistent themes have emerged from the surveys. There is general respect for psychiatry as a specialty, with all students agreeing on psychiatry placements being necessary, and psychiatrists being credible, ‘real doctors’. This may stand against the stigma experienced by psychiatrists.

Almost all students agree mental health conditions are effectively treatable (88%) – however zero students strongly agreed.

Some issues remain divisive for students: how mental health patients are treated, how comfortable they would feel seeking help for mental illness, or being left alone with a mental health patient.

This study had a small sample size (n=17), and would benefit from both more responses, as well as focus groups to gather qualitative data.

